# Indications of Depressive Symptoms During the COVID-19 Pandemic in Germany: Comparison of National Survey and Twitter Data

**DOI:** 10.2196/27140

**Published:** 2021-06-18

**Authors:** Caroline Cohrdes, Seren Yenikent, Jiawen Wu, Bilal Ghanem, Marc Franco-Salvador, Felicitas Vogelgesang

**Affiliations:** 1 Mental Health Research Unit Department of Epidemiology and Health Monitoring Robert Koch Institute Berlin Germany; 2 Symanto Research GmbH & Co KG Nuernberg Germany

**Keywords:** depressive symptoms, GEDA/EHIS survey, Twitter, COVID-19, pandemic, social contact ban, temporal progression, data correspondence, public mental health surveillance, depression, survey, social media, data, infodemiology, infoveillance, twitter, mental health, public health, surveillance, monitoring, symptom

## Abstract

**Background:**

The current COVID-19 pandemic is associated with extensive individual and societal challenges, including challenges to both physical and mental health. To date, the development of mental health problems such as depressive symptoms accompanying population-based federal distancing measures is largely unknown, and opportunities for rapid, effective, and valid monitoring are currently a relevant matter of investigation.

**Objective:**

In this study, we aim to investigate, first, the temporal progression of depressive symptoms during the COVID-19 pandemic and, second, the consistency of the results from tweets and survey-based self-reports of depressive symptoms within the same time period.

**Methods:**

Based on a cross-sectional population survey of 9011 German adolescents and adults (n=4659, 51.7% female; age groups from 15 to 50 years and older) and a sample of 88,900 tweets (n=74,587, 83.9% female; age groups from 10 to 50 years and older), we investigated five depressive symptoms (eg, depressed mood and energy loss) using items from the Patient Health Questionnaire (PHQ-8) before, during, and after relaxation of the first German social contact ban from January to July 2020.

**Results:**

On average, feelings of worthlessness were the least frequently reported symptom (survey: n=1011, 13.9%; Twitter: n=5103, 5.7%) and fatigue or loss of energy was the most frequently reported depressive symptom (survey: n=4472, 51.6%; Twitter: n=31,005, 34.9%) among both the survey and Twitter respondents. Young adult women and people living in federal districts with high COVID-19 infection rates were at an increased risk for depressive symptoms. The comparison of the survey and Twitter data before and after the first contact ban showed that German adolescents and adults had a significant decrease in feelings of fatigue and energy loss over time. The temporal progression of depressive symptoms showed high correspondence between both data sources (*ρ*=0.76-0.93; *P*<.001), except for diminished interest and depressed mood, which showed a steady increase even after the relaxation of the contact ban among the Twitter respondents but not among the survey respondents.

**Conclusions:**

Overall, the results indicate relatively small differences in depressive symptoms associated with social distancing measures during the COVID-19 pandemic and highlight the need to differentiate between positive (eg, energy level) and negative (eg, depressed mood) associations and variations over time. The results also underscore previous suggestions of Twitter data’s potential to help identify hot spots of declining and improving public mental health and thereby help provide early intervention measures, especially for young and middle-aged adults. Further efforts are needed to investigate the long-term consequences of recurring lockdown phases and to address the limitations of social media data such as Twitter data to establish real-time public mental surveillance approaches.

## Introduction

### Background

Worldwide, approximately 330 million people actively use Twitter at least once a month [1]. In Germany, as in other countries, the proportion of active Twitter users has increased over the past years and reached 17.1% of the population aged between 16 and 69 years in 2019 [2]. In light of this large group of users, web-based social communication platforms such as Twitter were proposed as potential sources of public health surveillance and early disease warning systems for the general population [3]. Recently, a review on the use of Twitter as a tool for health research revealed that approximately 23% of eligible studies were from the public health research realm [4]. The review showed that 26% of the studies used Twitter data for the surveillance of infectious diseases (eg, influenza [4]). However, a systematic investigation on the incremental value and reliability of Twitter as a tool for monitoring public *mental* health has not yet been conducted.

Worldwide, depressive disorders are among the most frequent mental diseases and among the three leading causes of nonfatal health loss and disability [5]. National health survey data similarly suggest a steady increase in depressive symptoms (eg, having little interest or pleasure in doing things and feeling down, depressed, or hopeless) over the past decades, with a recent prevalence of 10.4% among the adult population living in Germany [6]. Considering these findings and indications that mental health problems such as depressive symptoms may increase during epidemic or pandemic crises [7], it is of significance to strengthen the public mental health evidence in general and during the recent COVID-19 pandemic in particular.

### Twitter Communication on Depressive Symptoms

Research on social media content and its individual psychological functions and effects has significantly increased over the past 15 years [8]. Explorations of why individuals discuss mental health issues on Twitter and the content of these discussions have revealed that establishing a sense of community are among the most frequent themes, aside from seeking personal relief and expressing thoughts or feelings [9,10]. Digitally mediated communication offers easy and low-threshold possibilities to connect to others and to express and share experiences of psychosocial stressors [9,11]. This is of particular relevance since depressive disorders have been associated with experiences of social rejection [12] and feelings of social isolation [13]. Despite the strong evidence on the role of social support for the developmental course of depression [14], it has been pointed out how the mode of social interaction (ie, in-person or digitally mediated contact) can be decisive in this regard [15]. Given the recent ban on in-person social contact during the COVID-19 pandemic, it is assumed that social media use has become particularly relevant to the general population [16]. Thus, the analysis of trends in social media to draw conclusions on the mental health status and needs of a population could be an interesting tool for mental health provision and policy.

Furthermore, previous research indicated promising approaches to detect signs of depression in tweets [17] and predict the individual onset of a depressive episode based on supervised algorithms, including content and linguistic style analyses of tweets (eg, for negative affectivity [18]). Although much rarer than tracking or predicting the temporal progression of individuals’ mental health, researchers have monitored the course of mental health discussion in social media over time to draw conclusions on a current or prospective public mental health state. One example is a study from McClellan and colleagues [19] that showed how Twitter messages could be used to help to identify periods with more frequent content related to depression and suicide in social media and the association of this content with societal events such as World Suicide Prevention Day or the suicide of a prominent actor. The results demonstrated the potential of longitudinal social media analysis for identifying public mental health conditions that may otherwise be overlooked by mental health professionals [19]. However, solely computational approaches to capture depressive symptoms from tweets have also been criticized because of their disregard for the context of keywords and for theoretically driven approaches to identify keywords [20]. For instance, Mowery and colleagues [20] found that the majority of tweets containing predefined depression-related keywords were not necessarily indicative of depressive symptoms on closer examination. Hence, the implementation of reliable indicators for automated detection is essential for future investigations.

### Twitter Communication on Depressive Symptoms During the COVID-19 Pandemic

In the context of the current pandemic situation, social media has gained even more attention as a tool providing the opportunity to discuss diverse issues related to the SARS-CoV-2 virus and potentially relevant information for policy makers [21]. By analyzing the content of English language tweets and their number of likes from February to March 2020, Abd-Alrazaq and colleagues [21] drew conclusions on the scope and relative relevance of COVID-19–related topics. One major finding was that individuals most frequently liked tweets referring to economic loss. In addition, a few studies have examined the course of the development of COVID-19–related English tweets. For instance, Lwin et al [22] captured how feelings of fear decreased, whereas feelings of anger and sadness increased over time. Another analysis based on US tweets captured higher frequencies of stress, anxiety, and loneliness in the time period from March to May 2020 compared to the same period in 2019 [23]. Further results on the course of tweets, including tweets on depressive symptoms during the COVID-19 pandemic, are not yet available.

### Population-Based Evidence on Depressive Symptoms During the COVID-19 Pandemic

Although research on the psychological burden of quarantine and the psychological burden for high-risk groups such as medical staff during other pandemic situations has already yielded important results [24], findings on the general population under social contact bans during a pandemic are relatively rare. The first results from the COVID-19 pandemic based on a comparison of nationally representative US data from March to April 2020 and from 2017 to 2018 suggested a more than threefold higher prevalence of depression symptoms in the 2020 study period [25]. A large cross-sectional survey including 7236 Chinese residents conducted during the COVID-19 pandemic in February 2020 also indicated a relatively high prevalence of depressive symptoms at 20.1% [26,27]. A recent cross-sectional online study of 15,704 German residents conducted in the period from March to May 2020 also found an increased proportion of depressive symptoms at 14.3% [28] (as opposed to 10.4% as indicated by national survey data a few years before [6]). However, another representative German health survey found no significant differences in overall depressive symptomatology in 2020 compared to that in 2019 but indicated differences in single symptoms and specific time frames [29]. Additionally, it must be considered that most research has been cross-sectional, and investigations on the temporal progression and interrelatedness of depressive symptoms with social distancing measures in response to COVID-19 infection rates are not yet well established. Only a few results seem to underpin the assumption that strict stay-at-home orders are associated with decreasing mental health. One example is a Spanish survey that showed how depressive symptoms immediately increased after the stay-at-home order in March 2020 [30]. Thus, it may be advantageous to differentiate between different time periods and different symptoms to generate additional knowledge on the potential consequences of the current pandemic situation for the general population.

### This Study

Previous research has focused on the use of either cross-sectional surveys or social media data to investigate depressive symptoms. A systematic comparison of both data sources over time has not yet been performed. In this study, we aim to bridge this gap to gain knowledge on the prevalence of population-based depressive symptoms by examining the same standardized indicators but with different data sources. The central research questions are as follows: what is the developmental course of depressive symptoms in the general population before, during, and after the social contact ban decreed by the German government due to the COVID-19 pandemic and do tweets on depressive symptoms mirror population-based survey data?

Correspondingly, we aimed to, first, examine the temporal progression of depressive symptoms during the COVID-19 pandemic and, second, explore the consistency of the results between tweets and survey-based self-reports of depressive symptoms within the same time period. Based on these findings, we aimed to draw conclusions on how to interpret results from the two different data sources on the current mental health status of a certain society at a certain point in time and the comparability and validity of such results. These analyses focus on depressive symptoms during the beginning of the COVID-19 pandemic in Germany as observed through Twitter and national survey data. The results could facilitate the comprehensive and prompt understanding and monitoring of future variation in public mental health, with particular benefits for critical situations such as those during the COVID-19 pandemic.

## Methods

### Sample and Procedure

#### Survey

These analyses are based on two different samples. The first sample represents a subsample of 9011 survey participants (n=4783, 51.7% female; age groups: n=151, 4.5% 15-17 years; n=424, 8.4% 18-24 years; n=746, 13.7% 25-34 years; n=1616, 19.9% 35-49 years; and n=6074, 53.6% 50 years and older) drawn from a recently conducted German national health survey (GEDA/EHIS-19; April 2019 to August 2020; for further information, see Damerow et al [29]). The data collection period considered is calendar week 1 to calendar week 31 (January 1 to June 30) of 2020. During this period, the German government for the first time implemented social distancing measures to contain the spread of the SARS-CoV-2 virus (ban on major events on March 10, closing of borders on March 15, and ban on in-person contact on March 22). For a better understanding of the development of depressive symptoms during that time, we also examined a period of 11 weeks before and after the implementation of social distancing measures (the gradual reimplementation of the ban on contact started on May 2 with the reopening of hairdresser’s, food services, etc). Keeping in mind that the transition between strict lockdown and relaxation was not suddenly all-embracing, we first aggregated the data on a weekly basis and then summarized and labelled the three periods *before* (calendar week 1-11), *during* (calendar week 12-18), and *after* (calendar week 19-31) the contact ban.

#### Twitter

The second sample is constituted of Twitter users. We collected tweets from Germany (N=95,201) via the public Twitter application programming interface (API) by querying tweets that contained terms indicative of depressive symptoms as formulated in the standardized Patient Health Questionnaire (PHQ-8; see the following section) [31]. We then applied the Symanto proprietary linguistic analysis model with the collected tweets to identify the tweets with self-references (ie, first-person pronouns and verbs), which would indicate that the mentioned depressive symptom was related to the user themselves [32,33]. This Twitter corpus corresponded to the period from January 1 to July 30, 2020. In the same way as the survey data, the Twitter data were first aggregated on a weekly basis and then summarized into the three periods *before*, *during*, and *after* the contact ban. Two items (*less appetite* and *problems concentrating*) needed to be excluded from further analyses due to the relatively low frequencies of related tweets (<12 a day). The item *moving or speaking slowly* was excluded because there were problems correctly identifying this item in the tweets. For the remaining five items from the PHQ-8, we had 88,900 tweets (n=74,620, 83.9% female; age groups: n=11,385, 12.8% 10-17 years; n=51,272, 57.7% 18-24 years; n=11,485, 12.9% 25-34 years; n=9408, 10.6% 35-49 years; and n=5350, 6% 50 years and older).

Based on previous suggestions for how to improve health-related Twitter-based research [4], we also took demographic characteristics (region of residence, age, and sex) of the Twitter users into account if applicable. The number of tweets that included regional references was 60,321. Since stratification by the 16 German federal districts led to small sample sizes and loss of statistical power, we used the participants’ sex and age group as covariates for the regression modeling only. Descriptive statistics on the frequency of depressive symptoms grouped by sex, age group, and region can be obtained from Multimedia Appendix 1 (Tables S1 and S2).

The regional information was derived from publicly available location data on Twitter users. Twitter does not provide information on users’ age and gender. Thus, we used Symanto proprietary text analytics models [34,35] that predict age and gender based on the content of users’ tweets. These models were trained via deep learning technology [36] on millions of short texts by authors with known age and sex to identify distinct lexical and semantic patterns (eg, topics, word usage, and writing style) from each demographic group [37]. By applying the prediction models on the collected tweets, we inferred the age group and sex of the anonymous Twitter users to provide richer demographic information. The prediction models have been benchmarked against state-of-the-art models and have shown similar performance to public benchmarks for other languages, such as PAN'14 [38].

### Measurement of Depressive Symptoms

#### Survey

We analyzed the same five items from the PHQ-8 [31] as in the lexical Twitter analysis (see description in following section). These items indicated the experience of diminished interest, depressed mood, insomnia or hypersomnia, fatigue or loss of energy, and feelings of worthlessness or inappropriate guilt. To make the results more comparable to the frequencies derived from Twitter data, the original responses to the PHQ-8 that were provided on a 4-point rating scale, ranging from *not at all* (0) to *nearly every day* (3), were dichotomized into *not at all affected* (0) and *affected* (1), thereby yielded (relative) frequencies averaged across participants.

#### Twitter

**Figure 1:**
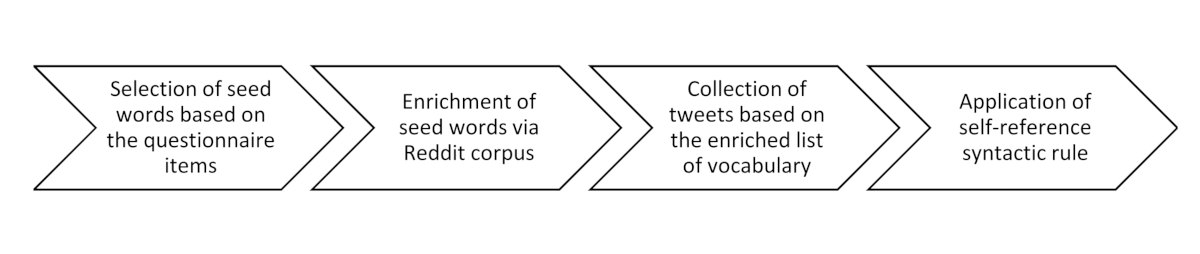
Flowchart summarizing the Twitter data collection process.

The terms used in the Twitter queries (previously mentioned) were curated to provide linguistic representations of the depressive symptoms in the Twitter analysis. The curation of this dictionary was based on a language processing methodology (see Figure 1 for an overview of the Twitter data collection process). We first manually produced a set of keywords (ie, seed words) by taking the items of the PHQ-8 as the basis. For instance, for the item that referred to depressed mood, three seed words were selected in this step: (1) “Niedergeschlagenheit” (dejection), (2) “Schwermut” (melancholy), and (3) “Hoffnungslosigkeit” (hopelessness). In the next step, to enrich the seed words, we extracted a corpus from a Reddit forum wherein the authors created and exchanged depression-related content in German [39]. This text corpus was then fed into the Symanto vocabulary refinement tool, which analyzes texts and represents each word with semantic dimensions using long short-term memory neural networks [40]. Using the seed words, this tool automatically identified words and phrases from the corpus that were semantically related to each of the seed words. For the item of depressed mood, for example, enrichment via the Reddit corpus revealed semantically related words to the three seed words, such as “aussichtslos” (hopeless), “ausweglos” (hopeless), “bedrückt” (depressed), “entmutigt” (discouraged), and “schlechte Stimmung” (bad mood). By doing so, we obtained an expanded vocabulary of depressive symptom keywords that were then used to collect the tweets. Having followed the tweet collection, we filtered out retweets so that only original tweets with the exact match of keywords (including hashtags and quoted tweets) would appear in the final data set. The final number of tweets among users varied between 1 and 10 tweets per week, with 96% (n=46,607) of the users accounting for 1 tweet only. Additionally, we applied syntactic rules to ensure that the keywords were mentioned in a self-reference manner. Example tweets that appeared in the final data set are provided in Textbox 1. Finally, we used the averaged frequency of daily tweets including depressive symptom keywords as a comparable measure to the (relative) frequencies derived from the survey.

**Example tweets from the final Twitter data set for each of the used Patient Health Questionnaire-8 items. Both the original German tweets and English translations are provided.**

**Diminished interest**

- “Ich habe keine Lust mehr auf Praktikum? So viel Heteronormativität ertrage ich nicht.”
- “I don't feel like doing an internship anymore? I can't stand that much heteronormativity.”

**Depressed mood**

- “Es ist aussichtslos. Ich bin ins Mark erschüttert.”
- “It is hopeless. I am shaken to the core.”

**Insomnia or hypersomnia**

- “Sind das nur Einschlafprobleme, oder leide ich unter Insomnie?”
- “Are these just problems falling asleep, or do I suffer from insomnia?”

**Fatigue or loss of energy**

- “Ja schon...Ich fühle mich immer so energielos.”
- “Yes I do...I always feel so low on energy.”

**Feelings of worthlessness**

- “Du denkst du könntest mich verletzen? Bro ich bin die Enttäuschung der Familie.”
- “You think you could hurt me? Bro I am the disappointment of the family.”

**Inappropriate guilt**

- “Ich denke nur darüber nach, wie es gefunden werden würde, wie sich meine Schwester fühlte. Schon mit dem Vater weg und jetzt bin ich tot. Die Szene von mir tot. Ich kann es nicht ertragen zu sterben, weil ich weiß, dass sie traurig sind”
- “I just think about how it would be found, how my sister felt. Already gone with the father and now I'm dead. The scene of me dead. I can't bear to die because I know they are sad.”

### Ethical Approvement and Data Availability

The GEDA/EHIS survey was conducted in accordance with the data protection provisions set out in the Federal Data Protection Act, and the Ethics Committee of the Charité Universitätsmedizin Berlin approved the study (No. EA2/070/19). The procedures used in this study adhere to the tenets of the Declaration of Helsinki. Participants gave their informed written consent. The Twitter data used in this study was collected via the Twitter Public API, and the use follows the Twitter Developer terms. No sensitive data is derived or inferred from individual Twitter users. Geo-data is solely used in aggregated format. A scientific use file of the GEDA/EHIS survey data will be available on request from the Health Monitoring Research Data Centre at the Robert Koch Institute in Berlin, Germany (email: fdz@rki.de) after release by the statistical office of the European Union (Eurostat). The full list of keywords and further information on text analytics technology applied in this project will be available on request at Symanto Research GmbH & Co KG (e-mail: info@symanto.com).

### Statistical Analyses

**Figure 2:**
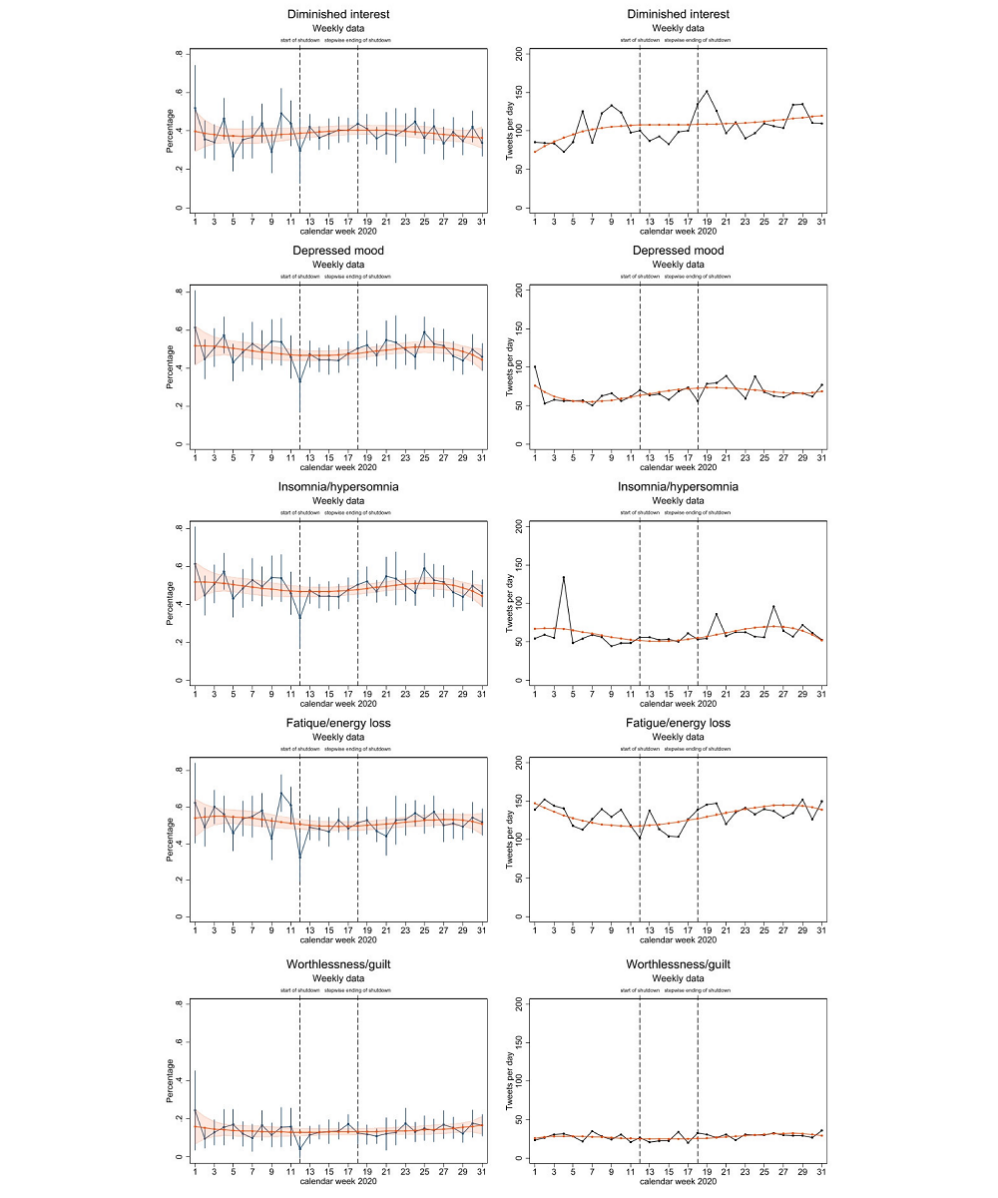
Temporal progression of five depressive symptoms in the survey data (on the left) and Twitter data (on the right) from January to August 2020 (calendar weeks 1-31). The dark gray line represents the weekly averaged frequency, and the orange line represents the smoothed progressions by fourth-degree polynomial.

We first conducted five logistic regression models (one for each PHQ item, with the PHQ items as the outcome variables) to predict the weekly number of depressive symptoms. The participant’s age, sex, and age × sex interactions were entered as control variables (model 1). As there were some weekly fluctuations in the survey data, these time estimates were smoothed by integrating the time variable *calendar week* as a fourth-degree polynomial (model 2). Both models were plotted together in one graph (Figure 2). To analyze whether there was a change in the number of depressive symptoms during the period of the first German social contact ban (calendar weeks 12-18), a third model (model 3) included a categorical variable dividing the survey into three time periods: before the contact ban (calendar weeks 1-11), during the contact ban, and after the stepwise relaxation of the contact ban (calendar weeks 19-31). We tested whether there was a significant change in the contact ban variable during these three time periods using an adjusted Wald test. Additionally, we pairwise compared the margins *before*, *during*, and *after the contact ban*. Models 1 to 3 were calculated using survey procedures to account for the complex sampling and weighting to balance the potential bias in significant demographic variables of the German population structure (eg, socioeconomic status, municipality, and migration background; see Damerow et al [29] for further details).

Following the same procedure used for the survey data, we aggregated the Twitter data on a weekly basis. Depressive symptoms were measured based on the number of tweets per day containing terms indicative of depressive symptoms, and we built a linear regression model (model 4) with age, sex, and age × sex interaction as the control variables. Again, these estimates were smoothed by integrating the calendar week in model 5 as a fourth-degree polynomial. In model 6, the influence of the contact ban was analyzed in the same way as in model 3.

Next, we investigated similarities in the time course of depressive symptoms between the Twitter and survey data. Due to the different scaling of frequencies in the Twitter data (absolute count) and survey data (binary variable indicating the presence or absence of symptoms for each survey respondent), and to the explorative character of these analyses, including possible violation of linearity, we calculated Spearman rank-order correlations for each of the five depressive symptoms. Correlations based on weekly aggregated smoothed margins are shown in Figure 2. The analyses were run with StataSE 15.1 (Stata Corp).

## Results

### Differences in Depressive Symptoms Before, During, and After the First Social Contact Ban

The differences in the frequencies of depressive symptoms among the three time periods largely followed the same pattern in the survey and Twitter data, as did the lower frequencies of insomnia and hypersomnia, fatigue and energy loss, and worthlessness and guilt, and the higher frequency of depressed mood during the contact ban than before and after the contact ban (Tables 1 and 2, Figure 2, and Table S3 in Multimedia Appendix 1). However, the differences among the time periods were significant only for fatigue and energy loss for both the survey and Twitter respondents and for worthlessness and guilt for the Twitter respondents. Whereas depressive symptoms after the contact ban were similar to those before the contact ban for the survey respondents, for several depressive symptoms, the frequency of symptoms after the contact ban was even higher than that before the ban for the Twitter respondents. Significant differences before and after the contact ban were found for diminished interest, depressed mood, and fatigue and energy loss (Tables 1 and 2, Table S3 in Multimedia Appendix 1, and Figure 2).

**Table 1:** Predictive margins of simple slopes resulting from regression analyses (model 3, survey data; model 6, Twitter data) for before, during, and after the contact ban.

Data			Diminished interest, margin (95% CI)		Depressed mood, margin (95% CI)		Insomnia/hypersomnia, margin (95% CI)		Fatigue/energy loss, margin (95% CI)		Worthlessness/guilt, margin (95% CI)
**Survey data**											
	Before (18.1%)	0.38 (0.35-0.42)		0.27 (0.24-0.30)		0.50 (0.46-0.53)		0.54 (0.51-0.57)		0.14 (0.11-0.16)	
	During (24.1%)	0.40 (0.38-0.43)		0.28 (0.25-0.30)		0.47 (0.44-0.50)		0.50 (0.47-0.52)		0.13 (0.11-0.15)	
	After (57.8%)	0.38 (0.36-0.41)		0.27 (0.25-0.29)		0.49 (0.47-0.52)		0.52 (0.49-0.54)		0.14 (0.13-0.16)	
**Twitter data**											
	Before (33.7%)	99.4 (90.3-108.5)		60.5 (55.8-65.1)		60.6 (51.6-69.7)		129.0 (122.1-135.8)		27.7 (25.7-29.7)	
	During (21.0%)	99.6 (88.5-110.8)		65.1 (59.3-70.8)		54.2 (42.6-65.7)		115.7 (107.3-124.1)		23.9 (21.3-26.5)	
	After (45.3%)	115.0 (106.8-123.3)		71.2 (67.0-75.5)		64.3 (55.8-72.8)		141.7 (135.7-147.8)		30.3 (28.5-32.0)	

**Table 2:** *P* values indicating the significance of the comparisons before, during, and after the contact ban resulting from the Wald test.

Data			Diminished interest, *P* value		Depressed mood, *P* value		Insomnia/hypersomnia, *P* value		Fatigue/energy loss, *P* value		Worthlessness/guilt, *P* value
**Survey data**											
	Before vs during	.34		.90		.23		.04		.74	
	Before vs after	.94		.75		.80		.21		.75	
	During vs after	.29		.61		.25		.29		.44	
**Twitter data**											
	Before vs during	.97		.22		.39		*.02^a^*		*.02*	
	Before vs after	*.01*		*.001*		.56		*.006*		.06	
	During vs after	*.03*		.09		.17		*<.001*		*<.001*	

^a^Italics indicate significant results at *P*<.05.

### Differences in Depressive Symptoms by Sex and Age Group

Between January and August 2020, the frequency of depressive symptoms among the survey respondents varied from 13.9% (n=1011) for feelings of worthlessness or inappropriate guilt to 51.6% (n=4472) for fatigue or loss of energy. Additionally, across the tweets, feelings of worthlessness were the least frequent depressive symptom (n=5103, 5.7%), and fatigue or loss of energy was the most frequent (n=31,005, 34.9%; see Table S1 in Multimedia Appendix 1). In general, female respondents reported depressive symptoms more frequently than male respondents, as indicated by the significant sex comparisons and age × sex interactions in Table S3 in Multimedia Appendix 1. The same result was found with both the Twitter and survey data. When we also considered the age of respondents, we observed that young adult women (younger than 25 years) most frequently reported depressive symptoms according to both the survey and Twitter data. Although depressive symptoms did not significantly differ between sexes among middle-aged adults, the frequency of symptoms was higher in female survey respondents aged 50 years than in male survey respondents, except for feelings of worthlessness and guilt. Correspondingly, the overall comparisons between age groups showed that older survey respondents (50 years and older) more frequently reported depressed mood and insomnia and hypersomnia than respondents younger than 18 years and reported diminished interest and worthlessness and guilt less frequently than age groups younger than 35 years and fatigue and energy loss less frequently than age groups between 25 and 49 years (Table S3 in Multimedia Appendix 1). The Twitter data similarly showed a higher frequency of depressed mood in older adults (50 years and older) than in respondents younger than 18 years. Another similarity was that Twitter respondents 50 years and older reported diminished interest less frequently than those aged 18 to 24 years and reported fatigue and energy loss less frequently than those aged 25 to 34 years. Contrary to the survey respondents, older adult Twitter users (50 years and older) reported feelings of worthlessness and guilt more frequently than all other age groups.

Additionally, the regional frequencies of depressive symptoms showed relatively good correspondence between the survey and Twitter data. The German districts of Nordrhein-Westfalen, Bayern, and some parts of Berlin had the highest number of overall depressive symptoms in the given time period. Notably, Nordrhein-Westfalen and Bayern also had the highest COVID-19 infection rates in Germany. The frequencies of depressive symptoms from the survey and Twitter data grouped by age group and sex as well as the regional frequencies grouped by the German federal districts can be obtained in Tables S1 and S2 in Multimedia Appendix 1.

### Associations in Depressive Symptoms Between Survey and Twitter Data Over Time

**Figure 3:**
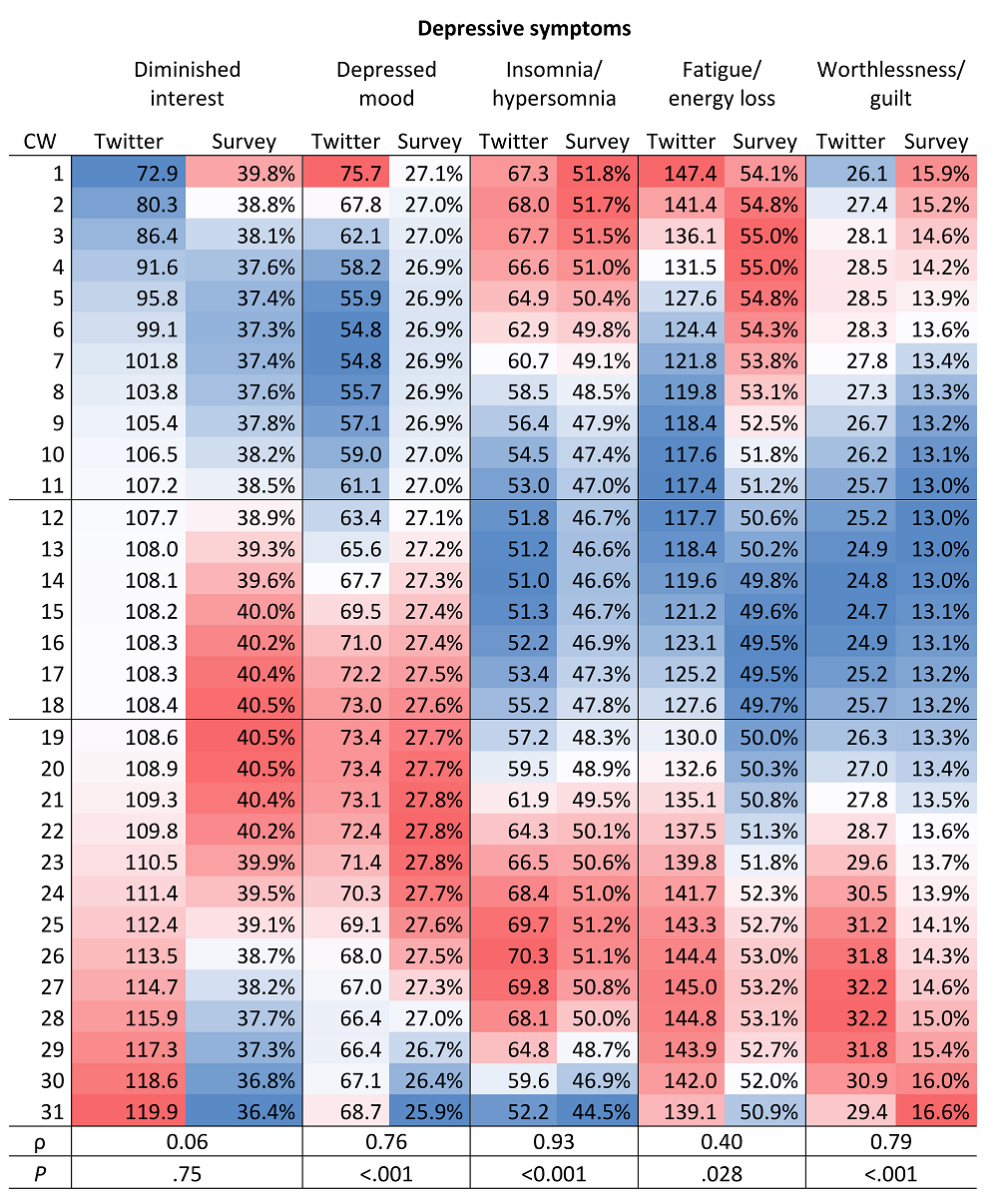
Predictive margins of the smoothed curves of depressive symptoms in the Twitter and survey data by CW from January to August 2020 and the Spearman rank correlation results. Color shading: white represents the mean values, dark blue indicates the lowest values, and dark red indicates the highest values. CW: 1-11 “before,” 12-18 “during,” and 19-31 "after" the German social contact ban. CW: calendar week.

Spearman rank-order correlations were calculated to assess the relationships between depressive symptoms based on the margins derived from smoothed curves over time (by calendar week) from the survey and Twitter data. The results showed moderate to strong positive correlations for depressed mood, insomnia and hyposomnia, fatigue and energy loss, and feelings of worthlessness and guilt (Figure 3). Diminished interest was not significantly correlated between the survey and Twitter data.

A closer look at the predictive margins of the weekly averaged and smoothed frequencies (model 4), as shown in Figures 2 and 3, indicated that the correspondence between depressive symptoms in the survey and Twitter data became even more evident when time shifts were taken into account. Whereas the courses of the symptoms of insomnia and hypersomnia and worthlessness and guilt were similar, reaching a minimum during the contact ban (as indicated by the blue color), depressed mood and fatigue and energy loss showed differences in temporal shifts in the Twitter and survey data. The frequencies of depressed mood and fatigue and energy loss in the Twitter data declined earlier before the contact ban and increased earlier during the contact ban than the frequencies of the same symptoms in the survey data.

## Discussion

In this study, we investigated indications of depressive symptoms before, during, and after the first social contact ban decreed by the German government due to the COVID-19 pandemic using survey and Twitter data from January to July 2020. We moreover differentiated between the age group and sex of the survey and Twitter respondents.

### Differences in Depressive Symptoms Before, During, and After the First Social Contact Ban

In contrast to previous indications of heightened depressive symptoms during the COVID-19 pandemic [25,27,28,30] and during lockdown periods [30], these findings reflect only a few significant differences in depressive symptoms before, during, and after the contact ban. In fact, the results indicate a temporary decrease in some depressive symptoms during the contact ban. The most distinct finding suggests that individuals had fewer problems related to fatigue and energy level during the contact ban than before or after the ban according to survey and Twitter data.

The explanations for the diverging results could be related to the fact that only a few previous studies have differentiated between diverse depressive symptoms or taken temporal fluctuations into account. Correspondingly, these findings are more in line with evidence on distinct mental health problems, showing a decrease in such problems immediately before the lockdown and the declaration of national emergencies in the United States (eg, fear [22]) and a steady increase thereafter (eg, stress and anger [22,23,40]). Similar temporal patterns of decreases and increases in depressive symptoms have been found in other challenging societal situations such as the global financial crisis in 2007 [41]. Within this context, a possible explanation for temporarily decreasing depressive symptoms over time was increased leisure time offering opportunities to foster social contact, pursue positive health behaviors (eg, a healthy diet and individual sports), or reduce work-related stress levels [42,43]. Moreover, emphases on collective experience and shared suffering as opposed to crises attributed to personal failure have been suggested as potential promoters of coping [41]. These assumptions are consistent with theories on coping with future stressful events (proactive coping [44]) and on expectancies of control [45]. The proactive coping approach postulates that coping in preparation for future stressors, for instance, to avoid or minimize negative effects, is associated with reduced stressor reactivity [46]; this approach is complemented by the idea that the controllability of a stressful situation can influence the efficacy of coping strategies and stress-related psychopathology [45]. In particular, an external locus of control (ie, a perception that something is outside of one’s personal power, such as in the hands of fate or the authorities) has been discussed as advantageous for coping success and mental health [47]. Thus, an anticipatory character and external locus of control combined with the collective experience of the actual pandemic crisis may have positively influenced depressive symptoms during the first German lockdown. The forced break and temporary release from several work- or social-related obligations may have been a relief and a chance to recover and gain energy for many people [43]. A few studies have shown positive behavioral adjustment during the COVID-19 pandemic in the general population, such as having a better diet [42], pursuing hobbies and interests, or spending more time outdoors [43].

### Differences in Depressive Symptoms by Sex and Age Group

However, there seem to be population groups that are not consistent with the general results and need specific attention. Evidence from economic crises, for instance, showed a high risk of mental burden for particular groups such as middle-aged men looking for work but not for the general population [41]. As opposed to (solely) economic crises, during the COVID-19 pandemic, it is not middle-aged men but young adult women who seem to be at high risk for depressive symptoms. This pattern was consistently evident in both the survey and Twitter data, and agrees with previous findings in Germany and in other countries [28,30,48] independent of the pandemic situation. It has already been discussed that the generally enhanced risk of depressive symptoms in females and young adults may have been amplified in light of the pandemic situation [26,49] and thus requires public attention and further exploration.

Additionally, the survey and Twitter data were consistent regarding the average relative frequency of depressive symptoms across the German federal districts. The two districts showing the comparatively highest numbers of depressive symptom reports were also those with the highest COVID-19 infection rates during the investigation period. Thus, one may conclude that Twitter data can reflect the cumulative occurrence of depressive symptoms within the general population based on demographic characteristics such as sex and regionality.

### Associations in Depressive Symptoms Between Survey and Twitter Data Over Time

A closer inspection of the temporal progression based on weekly averaged depressive symptoms from January to July 2020 emphasized similarities between the two investigated data sources. The correlations between the Twitter and survey data were moderate to high for depressed mood, insomnia and hypersomnia, fatigue and energy loss, and worthlessness and guilt. Interestingly, the results also suggest differences in reactions to the social contact ban, with the Twitter respondents seeming to react earlier than the survey respondents in terms of depressed mood and fatigue and energy loss. This observation may lead to the conclusion that Twitter users proactively react to the given circumstances; however, the reasons for temporal shifts are not yet well understood and require further investigation.

Despite the aforementioned similarities, there were also some differences between the survey and Twitter data that require further attention. Exceptions to the decreasing trend after the initiation of the lockdown period were found for the symptoms of depressed mood and diminished interest, which remained relatively constant in the survey respondents and continuously increased in the Twitter respondents, even after the relaxation of the social contact ban. This finding can be interpreted to indicate either that Twitter is an attractive tool for people experiencing depressed mood and diminished interest or that individuals are more willing to disclose information of that kind on Twitter than in a survey. The finding that the number of reports of depressive symptoms was estimated to be approximately 8% higher [20] in tweets than in responses to a national health survey conducted in 2019 [6] (18.2% vs 10.4%) supports the first assumption. On the other hand, motivations to tweet and to participate in a survey likely differ, but the evidence is inconclusive so far. Although there is evidence suggesting a disproportionate use of social media by individuals with mental problems or real-life relationship problems to overcome feelings of social isolation [11,50], other investigations have indicated a rather balanced use of social media in the general population compared to that in mentally ill individuals [51,52] and a lack of a relationship between social media use and (mental) health status [51,52]. Some findings even suggest a less active use of Twitter in individuals with diagnosed depression as compared to a nondepressed control group [17]. Within the context of the COVID-19 pandemic, the newly available leisure time during the social contact ban and the reduction of face-to-face contact may also have contributed to the higher use of Twitter as a medium to share thoughts and feelings. Previous results pointed toward the potential of social media to overcome loneliness and social isolation particularly in older adults [53] and have been discussed in terms of the pandemic situation [54]. Frequent social media use, however, was found to be associated with negative mental health status among Chinese citizens during the COVID-19 pandemic [55]. Further evidence is needed to get a clearer picture of the positive and negative effects of social media use and effects during the COVID-19 pandemic.

### Limitations

Apart from the potential benefit of Twitter as a tool to reflect public mental health conditions, there are some limitations of this study that have to be considered. First, although the proportion of Twitter users in the general population is constantly rising, we cannot rule out sampling and access bias. This study showed a disproportionate number of tweets across different population groups, reflecting an imbalance compared with other user statistics [56,57]. Consequently, the comparability of the survey and Twitter samples was limited. Second, the frequency of reported depressive symptoms cannot be interpreted as epidemiological prevalence rates because of the different coding and analysis approaches that were used in this study to enhance comparability. In addition, a general limitation of the study is related to the fact that depressive symptoms can be subject to seasonal variation [58], and this influence cannot be ruled out with this data. Accordingly, there is evidence suggesting that temporal, spatial, or geographical characteristics (eg, local weather or location-specific health characteristics) and other personal characteristics (eg, personality) can influence the content and sentiment of tweets [59-61]. Although this study considered the Twitter user’s location if applicable (ie, users revealed their living area) and the results indicate that depressive symptoms vary by local infection rates, the information is vague and further effort is needed to take other relevant and reliable characteristics into account. Furthermore, the lockdown may have affected the responsibilities of not only the Twitter users but also the survey respondents (eg, due to increased leisure time and better accessibility), which cannot be yet estimated.

### Conclusions

Overall, these results indicate rather small differences in depressive symptoms associated with social distancing measures during the COVID-19 pandemic and highlight the need to differentiate between positive (eg, energy level) and negative (eg, depressed mood) associations and variations over time. The inclusion of individual characteristics such as the age and sex of both survey and Twitter respondents helped to add new insight into the distribution of depressive symptom indications. As a result, we found exceptions with young adult women, who represented a high-risk group for depressive symptoms, and individuals living in federal districts with high infection rates.

These findings also underscore previous suggestions of the potential of Twitter data to help identify hot spots of declining and improving public mental health and to thereby help provide early intervention measures [23]. We were able to add knowledge to the consistency of findings based on two different data sources and its potential for public mental health monitoring. More precisely, we found considerable overlap between the Twitter and survey data, which offer notable entry points for monitoring public mental health, especially for young and middle-aged adults, but further investigation is also required. For instance, some of the key symptoms of depression (eg, energy loss) seemed to be relatively reliably detected in Twitter data, and these Twitter data showed high similarity with the corresponding survey data. Moreover, the temporal progression of depressive symptoms showed relatively high correspondence between the two data sources, providing notable indications of general trends in public mental health states for the time periods before, during, and after the social contact ban. The correspondence of these findings indicate how learning algorithms predicting socioeconomic characteristics and mental health states can continue to grow and help create opportunities to enhance information content and expand research applicability.

However, these results also highlight the need to consider limitations and challenges, and perform further validation to establish real-time public mental surveillance approaches [62]. For example, conclusions based on Twitter data will be limited since researchers are blind to several potentially confounding characteristics in Twitter data (eg, the basic population size). More differentiated analyses may allow more reliable approximation of Twitter user proportions and of mental health–related topics, for instance, by putting further emphasis on metadata like retweets, hashtags, or the number of followers [63]. Considering the sentiment of tweets may also contribute to a better approximation of the actual experience of mentioned depressive symptoms in users [17]. In sum, future investigations need to clarify the role of possible explanatory factors for differences between Twitter and survey data sources, such as the motivation or willingness to share and communicate sensitive mental health information in different contexts and the ability of individuals to remember and reflect such information. For instance, although depressed mood may be easy to communicate, feelings of worthlessness or guilt may not be.

## Appendix

Multimedia Appendix 1 Supplementary materials.

## Abbreviations

API: application programming interface
PHQ-8: patient health questionnaire
