# Supplementary material for: Indications of Depressive Symptoms During the COVID-19 Pandemic in Germany: Comparison of National Survey and Twitter Data
Source: JMIR Ment Health. 2021 Jun 18;8(6):e27140. doi: 10.2196/27140 (PMC8216331; doi:10.2196/27140)
Supplement: Multimedia Appendix 1 [file mental_v8i6e27140_app1.docx]

## Multimedia Appendix

Indications of Depressive Symptoms During the COVID-19 Pandemic in Germany: Comparison of National Survey and Twitter Data

Cohrdes, C., Yenikent, S., Wu, J., Ghanem, B., Franco-Salvador, M., & Vogelgesang, F.

Table S1. Frequency of Depressive Symptoms in the Survey (N=9,011) and Twitter Data (N=88,900) in Total and Grouped by Sex and Age Group from January to August 2020

|  | **Depressive symptoms** | | | | |
| --- | --- | --- | --- | --- | --- |
|  | Diminished interest | Depressed mood | Insomnia/ hypersomnia | Fatigue/ energy loss | Worthlessness/ guilt |
| **Survey data** (N participants unweighted, [% weighted]) | | | | | |
| Total | 3,049 [38.9] | 2,104 [27.2] | 4,395 [48.6] | 4,472 [51.6] | 1,011 [13.9] |
| Male | 1,251 [44.7] | 772 [40.0] | 1,784 [43.4] | 1,822 [44.0] | 367 [41.3] |
| Female | 1,798 [55.3] | 1,332 [60.0] | 2,611 [56.6] | 2,650 [56.0] | 644 [58.7] |
| <18 years | 73 [5.8] | 36 [4.3] | 51 [4.5] | 78 [4.5] | 39 [4.5] |
| 18-24 | 208 [11.0] | 139 [11.3] | 185 [8.4] | 237 [9.2] | 89 [8.4] |
| 25-34 | 299 [14.6] | 208 [14.7] | 316 [13.6] | 417 [15.0] | 123 [13.7] |
| 35-49 | 548 [18.7] | 370 [19.1] | 722 [19.9] | 850 [20.5] | 199 [19.9] |
| 50+ | 1,921 [49.8] | 1,351 [50.6] | 3,121 [53.6] | 2,890 [51.0] | 561 [53.6] |
| **Twitter data** (N tweets, %) | | |  |  |  |
| Total | 27,413 (30.8) | 16,069 (18.1) | 9,310 (10.5) | 31,005 (34.9) | 5,103 (5.7) |
| Male | 6,411 (23.4) | 3,271 (20.4) | 678 (7.3) | 2693 (8.7) | 1,227 (24.0) |
| Female | 21,002 (76.6) | 12,798 (79.6) | 8,632 (92.7) | 28,312 (91.3) | 3,876 (76.0) |
| <18 years | 4,009 (14.6) | 2,271 (14.1) | 1,22 (13.1) | 3,426 (11.1) | 457 (9.0) |
| 18-24 | 14,917 (54.4) | 8,361 (52.0) | 5,573 (59.9) | 19,652 (63.4) | 2,769 (54.3) |
| 25-34 | 3,951 (14.4) | 2,233 (13.9) | 958 (10.3) | 3,548 (11.4) | 795 (15.6) |
| 35-49 | 2,502 (9.1) | 1,976 (12.3) | 1,231 (13.2) | 3,204 (10.3) | 495 (9.7) |
| 50+ | 2,034 (7.4) | 1,228 (7.6) | 326 (3.5) | 1,175 (3.8) | 587 (11.5) |

Table S2. Frequency of Depressive Symptoms in the Survey (N=9,011) and Twitter Data (N=88,900) Grouped by the 16 German Federal States from January to August 2020

|  | **Depressive Symptoms** | | | | |
| --- | --- | --- | --- | --- | --- |
|  | **Diminished interest** | **Depressed mood** | **Insomnia/ hypersomnia** | **Fatigue/ energy loss** | **Worthlessness/ guilt** |
| **Survey data,** N unweighted [% weighted] | | | | | |
| Total | 3,049 | 2,104 | 4,395 | 4,472 | 1,011 |
|  |  |  |  |  |  |
| **Baden-Württemberg** | **383** | 247 | **558** | 573 | **134** |
|  | **[13.9]** | [13.4] | **[15.1]** | [14.6] | **[12.4]** |
| **Bayern** | **411** | **298** | **547** | **604** | **151** |
|  | **[15.9]** | **[16.2]** | **[15.1]** | **[15.7]** | **[16.3]** |
| **Berlin** | 321 | **250** | 475 | **507** | 123 |
|  | [5.3] | **[5.9]** | [5.1] | **[5.6]** | [5.9) |
| Brandenburg | 50 | 28 | 73 | 75 | 18 |
|  | [1.0] | [1.0] | [1.8] | [1.5] | [1.3] |
| Bremen | 17 | 11 | 22 | 29 | 6 |
|  | [1.0] | [0.7] | [0.7] | [1.1] | [0.5] |
| Hamburg | 29 | 22 | 49 | 53 | 10 |
|  | [1.1] | [1.2] | [1.4] | [1.4] | [1.7] |
| Hessen | 196 | 126 | 269 | 280 | 59 |
|  | [7.6] | [7.4] | [7.8] | [8.1] | [6.2] |
| Mecklenburg-Vorpommern | 37 | 32 | 60 | 52 | 12 |
|  | [1.8] | [2.2] | [1.7] | [1.4] | [2.1] |
| Niedersachsen | 213 | 155 | 332 | 327 | 78 |
|  | [9.0] | [9.8] | [9.0] | [8.9] | [11.6] |
| **Nordrhein-Westfalen** | **607** | **419** | **835** | **844** | **174** |
|  | **[25.0]** | **[24.1]** | **[23.1]** | **[2.4]** | **[21.7]** |
| Rheinland-Pfalz | 134 | 92 | 192 | 203 | 49 |
|  | [4.8] | [5.1] | [5.0] | [5.2] | [5.8] |
| Saarland | 396 | 257 | 623 | 585 | 112 |
|  | [3.1] | [3.0] | [3.2] | [2.9] | [2.8] |
| Sachsen | 87 | 55 | 134 | 129 | 28 |
|  | [3.8] | [3.5] | [4.1] | [3.8] | [4.3] |
| Sachsen-Anhalt | 54 | 36 | 77 | 65 | 15 |
|  | [2.8] | [2.6] | [2.7] | [2.4] | [2.4] |
| Schleswig-Holstein | 60 | 37 | 77 | 73 | 22 |
|  | [1.9] | [1.7] | [2.1] | [1.8] | [2.4] |
| Thüringen | 51 | 39 | 63 | 65 | 20 |
|  | [2.1] | [2.5] | [2.2] | [2.0] | [2.7] |
|  |  |  |  |  |  |
| **Twitter data, N (%)** |  |  |  |  |  |
| Total | 27,413  (30.8) | 16,069  (18.1) | 9,310  (10.5) | 31,005  (34.9) | 5,103  (5.7) |
|  |  |  |  |  |  |
| Unidentified region | 9,008 | 5,069 | 2,849 | 10,049 | 1,604 |
|  | (32.9) | (31.6) | (30.6) | (32.4) | (31.4) |
| Baden-Württemberg | 1,596 | 1,009 | 553 | 1,870 | 310 |
|  | (8.7) | (9.2) | (8.6) | (8.9) | (8.9) |
| **Bayern** | **2,176** | **1,229** | **721** | **2,359** | **385** |
|  | **(11.8)** | **(11.2)** | **(11.2)** | **(11.3)** | **(11.0)** |
| **Berlin** | **2,883** | **1,643** | **1,045** | **3,096** | **574** |
|  | **(15.7)** | **(14.9)** | **(16.2)** | **(14,8)** | **(16.4)** |
| Brandenburg | 127 | 78 | 55 | 144 | 22 |
|  | (0.7) | (0.7) | (0.9) | (0.7) | (0.6) |
| Bremen | 367 | 193 | 100 | 343 | 44 |
|  | (2.0) | (1.8) | (1.6) | (1.6) | (1.3) |
| Hamburg | 1,415 | 842 | 483 | 1,574 | 277 |
|  | (7.7) | (7.7) | (7.5) | (7.5) | (7.9) |
| Hessen | 1,460 | 913 | 522 | 1,687 | 281 |
|  | (7.9) | (8.3) | (8.1) | (8.0) | (8.0) |
| Mecklenburg-Vorpommern | 150 | 89 | 62 | 189 | 30 |
|  | (0.8) | (0.8) | (1.0) | (0.9) | (0.9) |
| Niedersachsen | 1,264 | 772 | 401 | 1,428 | 214 |
|  | (6.9) | (7.0) | (6.2) | (6.8) | (6.1) |
| **Nordrhein-Westfalen** | **4,861** | **2,958** | **1,726** | **5,512** | **933** |
|  | **(26.4)** | **(26.9)** | **(26.7)** | **(26.3)** | **(26.7)** |
| Rheinland-Pfalz | 496 | 303 | 185 | 698 | 102 |
|  | (2.7) | (2.8) | (2.9) | (3.3) | (2.9) |
| Saarland | 147 | 65 | 62 | 171 | 26 |
|  | (0.8) | (0.6) | (1.0) | (0.8) | (0.7) |
| Sachsen | 729 | 439 | 287 | 987 | 162 |
|  | (4.0) | (4.0) | (4.4) | (4.7) | (4.6) |
| Sachsen-Anhalt | 213 | 116 | 65 | 212 | 34 |
|  | (1.2) | (1.0) | (1.0) | (1.0) | (1.0) |
| Schleswig-Holstein | 243 | 189 | 106 | 330 | 48 |
|  | (1.3) | (1.7) | (1.6) | (1.6) | (1.4) |
| Thüringen | 278 | 162 | 88 | 356 | 57 |
|  | (1.5) | (1.5) | (1.4) | (1.7) | (1.6) |

*Note.* The German federal districts with the most frequent depressive symptoms are highlighted in boldface.

Table S3. Results from Logistic (Survey *N*=9,011; Model 1) and Multiple (Twitter *N*=64,073, Model 4) Regression Analyses Predicting Depressive Symptoms by Time Period (Before, During, and After the Social Contact Ban), Participant Age and Participant Sex

|  |  | **Depressive symptoms** | | | | | | | | | | | | | | | | | | | | | | | | | |
| --- | --- | --- | --- | --- | --- | --- | --- | --- | --- | --- | --- | --- | --- | --- | --- | --- | --- | --- | --- | --- | --- | --- | --- | --- | --- | --- | --- |
|  |  | **Diminished interest** | | |  | **Depressed mood** | | |  | **Insomnia/hypersomnia** | | | |  | **Fatigue/energy loss** | | | | |  | | **Worthlessness/guilt** | | | | | |
|  |  | ***OR*** | **95% CI** | ***P*** |  | ***OR*** | **95% CI** | ***P*** |  | ***OR*** | **95% CI** | | ***P*** |  | | ***OR*** | **95% CI** | | ***P*** |  | ***OR*** | | | **95% CI** | | ***P*** | |
| **Survey data^1^** | | | | | | | | | | | | | | | | | | | | | | | | | | |  |
| Intercept |  | 0.79 | 0.31-1.98 | .616 |  | 0.43 | 0.16-1.14 | .090 |  | 1.39 | 0.59-3.24 | .450 | |  | 1.17 | | | 0.45-3.04 | .750 |  | 0.19 | | 0.06-0.64 | | **.007** | | |
| **Sex** |  |  |  |  |  |  |  |  |  |  |  |  | |  |  | | |  |  |  |  | |  | |  | | |
| Male vs. female |  | 1.46 | 1.23-1.74 | **<.001** |  | 1.61 | 1.32-1.95 | **<.001** |  | 1.81 | 1.54-2.12 | **<.001** | |  | 1.61 | | | 1.37-1.88 | **<.001** |  | 1.45 | | 1.08-1.93 | | **.012** | | |
| **Age** |  |  |  |  |  |  |  |  |  |  |  |  | |  |  | | |  |  |  |  | |  | |  | | |
| 50+ vs. <18 |  | 1.76 | 1.02-3.03 | **.041** |  | 0.36 | 0.15-0.88 | **.026** |  | 0.54 | 0.30-0.98 | **.042** | |  | 1.04 | | | 0.6-1.79 | .894 |  | 2.13 | | 1.01-4.47 | | **.046** | | |
| 50+ vs. 18-24 |  | 1.98 | 1.38-2.84 | **.000** |  | 1.45 | 0.94-2.23 | .096 |  | 0.88 | 0.61-1.27 | .507 | |  | 1.23 | | | 0.86-1.77 | .261 |  | 3.12 | | 1.93-5.03 | | **.000** | | |
| 50+ vs. 25-34 |  | 1.70 | 1.25-2.31 | **.001** |  | 1.41 | 0.99-1.99 | .054 |  | 0.92 | 0.68-1.24 | .598 | |  | 1.66 | | | 1.24-2.23 | **.001** |  | 1.94 | | 1.21-3.11 | | **.006** | | |
| 50+ vs. 35-49 |  | 1.15 | 0.88-1.51 | .314 |  | 1.22 | 0.89-1.67 | .209 |  | 0.99 | 0.78-1.27 | .949 | |  | 1.45 | | | 1.13-1.85 | **.003** |  | 1.45 | | 0.93-2.28 | | .104 | | |
| **Age^1^ × sex** | |  |  |  |  |  |  |  |  |  |  |  | |  |  | | |  |  |  |  | |  | |  | | |
| <18 male × female |  | 1.13 | 0.54-2.37 | .748 |  | 5.28 | 1.88-14.87 | **.002** |  | 0.86 | 0.39-1.89 | .707 | |  | 0.99 | | | 0.47-2.1 | .984 |  | 2.26 | | 0.9-5.69 | | .083 | | |
| 18-24 × male vs. female |  | 0.89 | 0.53-1.51 | .673 |  | 1.34 | 0.76-2.38 | .314 |  | 0.71 | 0.42-1.19 | .195 | |  | 1.23 | | | 0.73-2.07 | .445 |  | 0.71 | | 0.37-1.36 | | .298 | | |
| 25-34 × male  vs. female |  | 0.56 | 0.36-0.88 | **.012** |  | 0.77 | 0.47-1.25 | .285 |  | 0.52 | 0.34-0.81 | **.004** | |  | 0.70 | | | 0.45-1.08 | .109 |  | 0.97 | | 0.52-1.79 | | .917 | | |
| 35-49 × male vs. female |  | 0.80 | 0.56-1.14 | .218 |  | 0.74 | 0.5-1.11 | .148 |  | 0.63 | 0.45-0.87 | **.006** | |  | 0.69 | | | 0.5-0.95 | **.023** |  | 0.90 | | 0.52-1.57 | | .718 | | |
| **Twitter data** | | | | | | | | | | | | | | | | | | | | | | | | | | |  |
|  |  | ***B*** | **95% CI** | ***P*** |  | ***B*** | **95% CI** | ***P*** |  | ***B*** | **95% CI** | ***P*** | |  | ***B*** | | | **95% CI** | ***P*** |  | ***B*** | | **95% CI** | | ***P*** | | |
| Intercept |  | 28.78 | -9.99-67.55 | .146 |  | 62.28 | 41.84-82.73 | **<.001** |  | 7.94 | -36.35-52.22 | .725 | |  | 25.17 | | | -3.81-54.15 | .089 |  | 15.96 | | 7.53-24.4 | | **<.001** | | |
| **Sex** |  |  |  |  |  |  |  |  |  |  |  |  | |  |  | | |  |  |  |  | |  | |  | | |
| Male vs. female |  | -23.91 | -48.09-0.26 | .053 |  | -8.07 | -20.6-4.46 | .207 |  | -2.64 | -33.12-27.84 | .865 | |  | 14.74 | | | -3.46-32.93 | .112 |  | -7.14 | | -12.58--1.7 | | **.010** | | |
| **Age** |  |  |  |  |  |  |  |  |  |  |  |  | |  |  | | |  |  |  |  | |  | |  | | |
| 50+ vs. <18 |  | -19.28 | -43.66-5.09 | .121 |  | -13.96 | -27.32--0.61 | **.040** |  | 1.17 | -38.5-40.83 | .954 | |  | -2.12 | | | -21.72-17.47 | .832 |  | -8.49 | | -15.32--1.66 | | **.015** | | |
| 50+ vs. 18-24 |  | 27.08 | 3.42-50.75 | **.025** |  | 12.47 | 0.40-24.55 | **.043** |  | 13.70 | -18.72-46.13 | .407 | |  | 21.82 | | | 3.86-39.77 | **.017** |  | -2.06 | | -7.11-3 | | .425 | | |
| 50+ vs. 25-34 |  | -8.29 | -32.19-15.61 | .496 |  | -9.54 | -22.08-2.99 | .136 |  | -0.06 | -40.63-40.52 | .998 | |  | -2.71 | | | -22.19-16.76 | .785 |  | -8.78 | | -14.17--3.38 | | **.001** | | |
| 50+ vs. 35-49 |  | -10.57 | -34.38-13.24 | .384 |  | -2.89 | -15.3-9.52 | .648 |  | -3.24 | -39.74-33.26 | .862 | |  | 13.13 | | | -5.83-32.08 | .175 |  | -7.46 | | -12.96--1.97 | | **.008** | | |
| **Age^1^ × sex** | |  |  |  |  |  |  |  |  |  |  |  | |  |  | | |  |  |  |  | |  | |  | | |
| <18 male × female |  | 112.68 | 78.47-146.9 | **<.001** |  | 65.96 | 47.85-84.08 | **<.000** |  | 28.23 | -16.95-73.41 | .221 | |  | 86.33 | | | 60.33-112.33 | **<.001** |  | 13.20 | | 4.52-21.89 | | **.003** | | |
| 18-24 × male vs. female |  | 426.73 | 393.02-460.44 | **<.001** |  | 237.05 | 219.85-254.25 | **<.000** |  | 170.36 | 131.35-209.38 | **<.001** | |  | 645.26 | | | 620.48-670.05 | **<.001** |  | 78.90 | | 71.73-86.06 | | **<.001** | | |
| 25-34 × male  Vs. female |  | 87.16 | 53.28-121.03 | **<.001** |  | 53.24 | 35.72-70.76 | **<.001** |  | 21.89 | -24.18-67.97 | .351 | |  | 92.93 | | | 67.02-118.84 | **<.001** |  | 20.96 | | 13.49-28.43 | | **<.001** | | |
| 35-49 × male vs. female |  | 37.47 | 3.59-71.34 | **.030** |  | 30.95 | 13.5-48.4 | **.001** |  | 36.27 | -6.4-78.93 | .096 | |  | 49.82 | | | 24.3-75.35 | **<.001** |  | 11.79 | | 3.96-19.63 | | **.003** | | |

*Notes.* ^1^ Reference category: 50+ male × 50+ female. OR=odds ratio, CI= confidence interval, B=unstandardized beta coefficient. Significant results at *p* <.05 are highlighted in boldface
